# Comorbidity burden at dialysis initiation and mortality: A cohort study

**DOI:** 10.1186/s40697-015-0068-3

**Published:** 2015-09-08

**Authors:** Alwyn T Gomez, Bryce A Kiberd, J Patrick Royston, Talal Alfaadhel, Steven D Soroka, Brenda R Hemmelgarn, Karthik K Tennankore

**Affiliations:** Faculty of Medicine, Dalhousie University, Halifax, NS Canada; Department of Medicine (Division of Nephrology), Dalhousie University, Halifax, NS Canada; Nova Scotia Health Authority, 5820 University Avenue, Halifax, NS Canada B3H 1V8; MRC Clinical Trials unit at UCL, London, UK; Department of Medicine, University of Calgary, Calgary, AB Canada; Department of Community Health Sciences, University of Calgary, Calgary, AB Canada

## Abstract

**Background:**

A high level of comorbidity at dialysis initiation is associated with an increased risk of death. However, contemporary assessments of the validity and prognostic value of comorbidity indices are lacking.

**Objectives:**

To assess the validity of two comorbidity indices and to determine if a high degree of comorbidity is associated with mortality among dialysis patients.

**Design:**

Cohort study.

**Setting:**

QEII Health Sciences Centre (Halifax, Nova Scotia, Canada).

**Patients:**

Incident, chronic dialysis patients between 01 Jan 2006 and 01 Jul 2013.

**Measurements:**

*Exposure:* The Charlson Comorbidity Index (CCI) and End-Stage Renal Disease Comorbidity Index (ESRD-CI) were used to classify individual comorbid conditions into an overall score. Comorbidities were classified using patient charts and electronic records.

*Outcome:* All-cause mortality.

*Confounders:* Patient demographics, dialysis access, cause of ESRD and baseline laboratory data.

**Methods:**

Regression coefficients were estimated on the CCI and ESRD-CI. Discrimination for death was assessed using Harrell’s c-index. Adjusted Cox proportional hazard models were used to calculate relative hazards and 95 % confidence intervals for each category of the CCI and ESRD-CI.

**Results:**

The cohort consisted of 771 ESRD patients from 01 Jan 2006 to 01 Jul 2013. Most were male (62 %) and Caucasian (91 %). The cohort had a high proportion of diabetes (48 %), history of previous myocardial infarction (31 %) and heart failure (22 %). Regression coefficients on the CCI and ESRD-CI were 0.55 and 0.52, respectively. The *c*-index, for the prediction of death, was 0.61 for the CCI and 0.63 for the ESRD-CI. ESRD-CI scores of 4, 5 and ≥6 were associated with a similar mortality risk (adjusted relative hazard of 1.95, 1.89 and 1.99, respectively). There was a small increased mortality risk for CCI scores of 4, 5 and ≥6 (adjusted relative hazard of 1.86, 2.38 and 2.71, respectively).

**Limitations:**

Classification of comorbidities for each patient was determined by clinical impression.

**Conclusions:**

The CCI and ESRD-CI have a limited ability to discriminate mortality risk for incident dialysis patients. Acknowledging the frequency with which they are used, this study emphasizes the need to re-examine the usefulness of previously derived comorbidity indices in contemporary dialysis cohorts.

## Résumé

**Contexte:**

Un taux élevé de comorbidité en début de dialyse est lié à un risque accru de mort. Toutefois, rares sont les évaluations récentes de la validité et de la valeur pronostique des indices de comorbidité.

**Objectifs:**

Vérifier la validité de deux indices de comorbidité et déterminer la relation entre un taux élevé de comorbidité et le taux de mortalité chez les patients dialysés.

**Type d’étude:**

Étude de cohorte.

**Contexte:**

Centre des sciences de la santé QEII (Halifax, Nouvelle-Écosse, Canada).

**Participants:**

Patients incidents en dialyse chronique du 1^er^ janv. 2006 au 1^er^ juil. 2013.

**Mesures:**

*Exposition:* L'indice de comorbidité Charlson (CCI) et l'indice de comorbidité au stade terminal d'insuffisance rénale (ESRD-CI) ont servi à la mesure de la comorbidité, à la lumière des fiches et des dossiers électroniques des patients.

*Résultat:* Taux de mortalité, toutes causes confondues.

*Facteurs de confusion:* caractéristiques sociodémographiques des patients, accès à la dialyse, cause de l'insuffisance rénale terminale (IRT) et données de référence du laboratoire.

**Méthodes:**

On a procédé à l'estimation des coefficients de régression du CCI et de l'ESRD-CI, puis à l'évaluation du seuil de mortalité à l'aide de l'indice C de Harrell. On a enfin utilisé des modèles des risques proportionnels de Cox ajustés afin de calculer les risques relatifs et les intervalles de confiance à 95 % pour chaque catégorie du CCI et de l'ESRD-CI.

**Résultats:**

La cohorte comprenait 771 patients en IRT du 1^er^ janv. 2006 au 1^er^ juil. 2013. La plupart étaient des hommes (62 %) de race blanche (91 %). On y trouvait une proportion élevée de diabète (48 %), d'infarctus du myocarde antérieur (31 %), et d'insuffisance cardiaque (22 %). Les coefficients de régression du CCI et de l'ESRD-CI indiquaient 0,55 et 0,52, respectivement. L'indice C du risque de décès était de 0,61 pour le CCI et de 0,63 pour l'ESRD-CI. Pour ce dernier indice, des valeurs de 4, 5 et 6 ou plus étaient liées à un risque de mortalité équivalent (risque relatif ajusté de 1,95, de 1,89 et de 1,99, respectivement). On a noté une légère augmentation du risque de mortalité pour les valeurs du CCI de 4, 5 et 6 ou plus (risque relatif ajusté de 1,86, de 2,38 et de 2,71, respectivement).

**Limites:**

Le classement des comorbidités de chaque patient était déterminé par opinion clinique.

**Conclusions:**

Le CCI et l'ESRD-CI sont limités en ce qui a trait à la capacité de déterminer le risque de mortalité chez une population incidente dialysée. En regard de la fréquence d’utilisation de la dialyse, la présente étude souligne le besoin de réévaluer l'utilité des indices de comorbidité précédemment dérivés des récentes cohortes dialysées.

## What was known before

The Charlson Comorbidity Index (CCI) and End-Stage Renal Disease Comorbidity Index (ESRD-CI) are commonly used in studies of dialysis patients, but assessments of their validity are lacking.

## What this adds

Both indices had a limited ability to discriminate mortality risk in this study emphasizing that they may not be the best method of risk adjustment in contemporary dialysis cohorts.

## Introduction

In patients with end-stage renal disease (ESRD), the presence of comorbid conditions has been shown to have a negative impact on survival [1–3]. A commonly used approach for summating individual conditions into an overall “score” of comorbidity for risk stratification is calculation of the Charlson Comorbidity Index (CCI) [4]. The CCI was initially derived in a cohort of 559 patients, and tested in a second cohort of 680 patients followed for 10 years [4]. In the original study, 19 comorbid conditions were evaluated in a Cox proportional hazards model. Point scores were assigned to each comorbidities hazard ratio depending on the value. The sum of the points equalled a given individuals’ overall CCI score. A higher CCI score is associated with an increased mortality risk in ESRD patients [5–12], however the CCI does have limitations when applied to ESRD patients. The inclusion of renal disease as one of the component comorbidities is redundant and medical advances since the development of the CCI have changed the prognosis of some of the individual comorbid conditions within the index [13].

There have been a number of additional comorbidity indices that have been created for ESRD patients [1, 14–16], including the End Stage Renal Disease Comorbidity Index (ESRD-CI) which avoids some of the limitations associated with the original CCI [15]. Having been designed as an adaptation of the CCI, the ESRD-CI was developed in a cohort of 237 incident dialysis patients [15]. 15 of the 19 conditions in the CCI were evaluated in a multivariable Cox survival analysis. Similar to the CCI, point scores were assigned to each condition’s hazard ratio and summed for each individual in the derivation cohort. In the model derivation study the ESRD-CI had slightly better performance characteristics compared to the CCI in the tested population (c-statistic of 0.73 versus 0.72) [15].

While both the CCI and ESRD-CI are frequently used for risk adjustment in studies of dialysis patients [17–22], only a few studies have attempted to validate either index [15, 23, 24]. In addition, these validation studies have limitations including incomplete inclusion of all necessary comorbid conditions, and validation techniques that are not specific to time-to-event analyses [15, 23, 24]. Finally, validation in a more recent era-cohort (acknowledging that patient characteristics, disease prevalence and outcome after dialysis initiation may differ from those in older cohorts) has not been conducted in many studies. A lack of validity/limited prognostic ability of either index will emphasize the need to re-examine the usefulness of previously derived comorbidity indices in contemporary dialysis cohorts.

Therefore, the purpose of this study was to assess the validity of the CCI and ESRD-CI in a contemporary cohort of ESRD patients, and to determine if a high degree of comorbidity was independently associated with mortality. We hypothesized that both indices would have a reduced level of discrimination compared to the derivation studies, but that increased comorbidity burden would be associated with a higher risk of death for dialysis patients.

## Methods

### Population

We conducted a cohort study of incident, adult (≥18 years) chronic dialysis patients in a large tertiary care institute between 1 Jan 2006 and 01 Jul 2013. Follow-up for patients began at the initial start date for their dialysis.

### Exposure definition

Comorbidity data was collected at the start of dialysis in all patients in a prospective manner, using documentation in patient charts (dating back to the first nephrology visit) and electronic records by the patients’ primary nephrologist. Comorbidities were subsequently verified in all patients by two nephrologists (K.T. and B.K.) and one nephrology trainee (T.A.). All 19 individual comorbid conditions in the CCI were collected at the time of dialysis initiation and scored according to the CCI derivation study. ESRD-CI scores were retrospectively calculated by re-scoring the comorbidities comprising the ESRD-CI based on the derivation paper. ESRD-CI scores were analyzed as ordinal variables and after categorization into six groups to be consistent with the derivation paper (using scores of 0/1, 2, 3, 4, 5 and ≥6) [15]. The CCI was analyzed both as an ordinal variable and in categories (2, 3, 4, 5 and ≥6). Since all patients in our dataset had ESRD, the lowest possible CCI score was 2.

In addition to the CCI and ESRD-CI scores, demographic data (age, race, gender), dialysis access (central venous catheter or arteriovenous fistula) type of dialysis modality (peritoneal or hemodialysis), cause of ESRD (diabetes, glomerulonephritis, hypertension, other) and baseline laboratory data (hemoglobin, phosphate, estimated glomerular filtration rate and albumin) were collected on all patients at the start of dialysis using a combination of electronic records and paper chart review.

### Outcome

The primary outcome was all cause mortality after dialysis initiation. Administrative censoring was imposed on 01 Jan 2014. Patient survival was censored at the date of transplantation.

### Statistical analysis

Descriptive statistics for baseline characteristics of the study cohort were reported as counts with proportions, mean with standard deviation and median with interquartile range for categorical, normally distributed continuous and non-normally distributed continuous variables, respectively.

External validation of the CCI and ESRD-CI followed the methods previously described by Royston *et al.* based on availability of data in the original derivation studies [25]:

1. Regression coefficients were estimated on the CCI and ESRD-CI (defined as the precise CCI and precise ESRD-CI).
2. Regression coefficients were also estimated on the categorical CCI (2, 3, 4, 5 and 6+) and ESRD-CI (0/1, 2, 3, 4, 5 and 6+) as a secondary analysis (defined as the categorical CCI and categorical ESRD-CI).
3. Discrimination (defined as the level of concordance between the risk predicted by a model and the rate of events experienced [25]) was assessed for the precise and categorical CCI as well as the precise and categorical ESRD-CI using Harrell’s *c*-index. Harrell’s c-index assesses the fraction of all possible pairings of patients in which the predictions and outcomes are concordant [26]. Scores range from 0.5 (no discrimination), to 1.0 (perfect discrimination). As a reference, the CHADS2 score for atrial fibrillation stroke risk has a reported *c*-index of 0.683 [27].
4. Kaplan-Meier Survival curves were plotted for each category of CCI/ESRD-CI and discrimination was also visually assessed according to the ordering and separation of the curves.

Cox proportional hazard models were used to calculated relative hazards and 95 % confidence intervals for each category of the CCI and ESRD-CI. Proportionality of hazards was assessed using Schoenfeld Residuals. Multivariable models included variables based on clinical judgment and those derived from the literature as being associated with mortality in studies of dialysis patients including age [28], gender [29], Caucasian versus non-Caucasian race [28, 30], dialysis modality [31], cause of ESRD, albumin [32], hemoglobin [33], phosphate [34, 35] and modification of diet in renal disease (MDRD) estimated glomerular filtration rate [36]. A two sided P value of <0.05 was the threshold for statistical significance. Approval to conduct this study was granted by our institutional research ethics board (Nova Scotia Health Authority, CDHA-RS/2014-288). All analyses were conducted using Stata version 12.0, College Station, TX, USA.

## Results

### Baseline characteristics

The cohort consisted of 771 ESRD patients from 01 Jan 2006 to 01 Jul 2013. Baseline characteristics of the cohort are noted in Table 1. The majority of patients were male (62 %) and Caucasian (91 %). Common comorbidities included diabetes (48 %), previous myocardial infarction (31 %), congestive heart failure (22 %) and peripheral vascular disease (20 %). The median CCI score was 4 (Q1-Q3: 3–6), and the median ESRD-CI Score was 2 (Q1-Q3: 0–4).

**Table 1:** Baseline characteristics of the cohort

Demographics	
Age (mean years ± SD)	62.6 ± 15.1
Male, n (%)	479 (62.1)
Caucasian, n (%)	702 (91.1)
Cause of ESRD, n (%)	
Diabetes	235 (30.5)
Other	158 (20.5)
Ischemic/Hypertension	138 (17.9)
Unknown	94 (12.2)
Glomerulonephritis	86 (11.2)
Polycystic kidney disease	60 (7.8)
^a^Select Comorbidities, n (%)	
Diabetes with complications	286 (37.1)
Myocardial infarction	236 (30.6)
Congestive heart failure	172 (22.3)
Peripheral vascular disease	154 (20.0)
Chronic lung disease	129 (16.7)
Cerebrovascular disease	93 (12.1)
Diabetes without complications	81 (10.5)
Neoplasia	50 (6.5)
Peptic ulcer disease	49 (6.4)
Laboratory	
MDRD GFR [median mL/min/1.73 m^2^ (Q1-Q3)]	8 (6–10)
Albumin (mean g/dL ± SD)	3.14 ± 0.65
Phosphate, n = 768 [median mg/dL (Q1-Q3)]	5.9 (5.0-7.4)
Hemoglobin, n = 770 [median g/dL (Q1-Q3)]	9.7 (8.6-10.9)
Dialysis Access, n (%)	
Hemodialysis with central venous catheter	410 (53.2)
Hemodialysis with arteriovenous fistula	194 (25.2)
Peritoneal dialysis	167 (21.7)
End Stage Renal Disease Comorbidity Index, n (%)	
Index score of ≤1	253 (32.8)
Index score of 2	161 (20.9)
Index score of 3	62 (8.0)
Index score of 4	92 (11.9)
Index score of 5	59 (7.7)
Index score of ≥6	144 (18.7)
Charlson Comorbidity Index, n (%)	
Index score of ≤1	0 (0)
Index score of 2	189 (24.6)
Index score of 3	100 (13.0)
Index score of 4	152 (19.7)
Index score of 5	120 (15.6)
Index score of ≥6	210 (27.2)

^a^Remaining comorbid conditions comprising the CCI and ESRD-CI (rheumatological, dementia, mild liver disease, moderate/severe liver disease, metastatic disease, leukemia, lymphoma, human immunodeficiency virus) were present in less than 5 % of the dialysis population at dialysis initiation)

### Validation

**Fig. 1:**
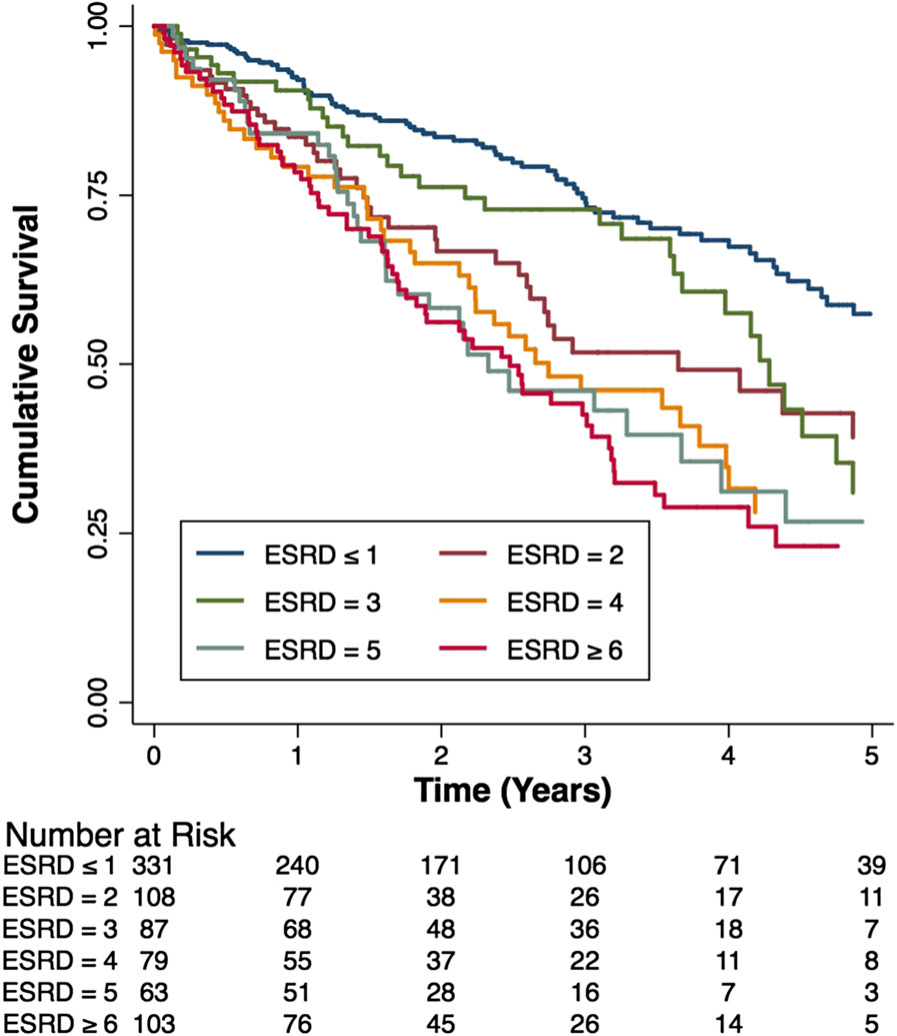
Kaplan-Meier survival curves for time to death stratified by ESRD-CI Score groups

**Fig. 2:**
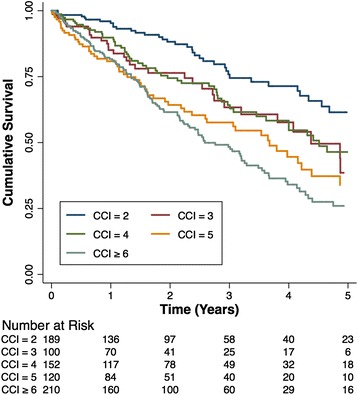
Kaplan-Meier survival curves for time to death stratified by CCI Score groups

Cox regression on the precise CCI and ESRD-CI revealed coefficients of 0.55 (SE 0.08) and 0.52 (SE 0.07), respectively (Table 2). Cox regression on the categorical CCI and ESRD-CI revealed similar coefficients (0.56 and 0.52, respectively). The *c*-index was 0.61 (SE 0.02) for both the precise and categorical CCI, and 0.63 (SE 0.02) and 0.62 (SE 0.02) for the precise and categorical ESRD-CI (Table 2). Kaplan-Meier survival curves for each ESRD-CI score cut-off are displayed in Fig. 1 (Log-rank P < 0.001). There was separation of the curves for patients with a high versus low score (6^+^ versus 0/1 or 2). However, discordance was observed for patients with an intermediate score. A similar finding was noted for the CCI, however, there was slightly more separation between the curves in an incremental fashion based on score (Fig. 2).

**Table 2:** Cox regression coefficients and c-index for precise and categorical CCI/ESRD-CI

Index	Regression coefficient [95 % CI]	c-index
Precise CCI	0.55 [0.38-0.71]	0.61
Categorical CCI	0.56 [0.39-0.72]	0.61
Precise ESRD-CI	0.52 [0.38-0.66]	0.63
Categorical ESRD-CI	0.52 [0.38-0.66]	0.62

### Mortality

**Fig. 3:**
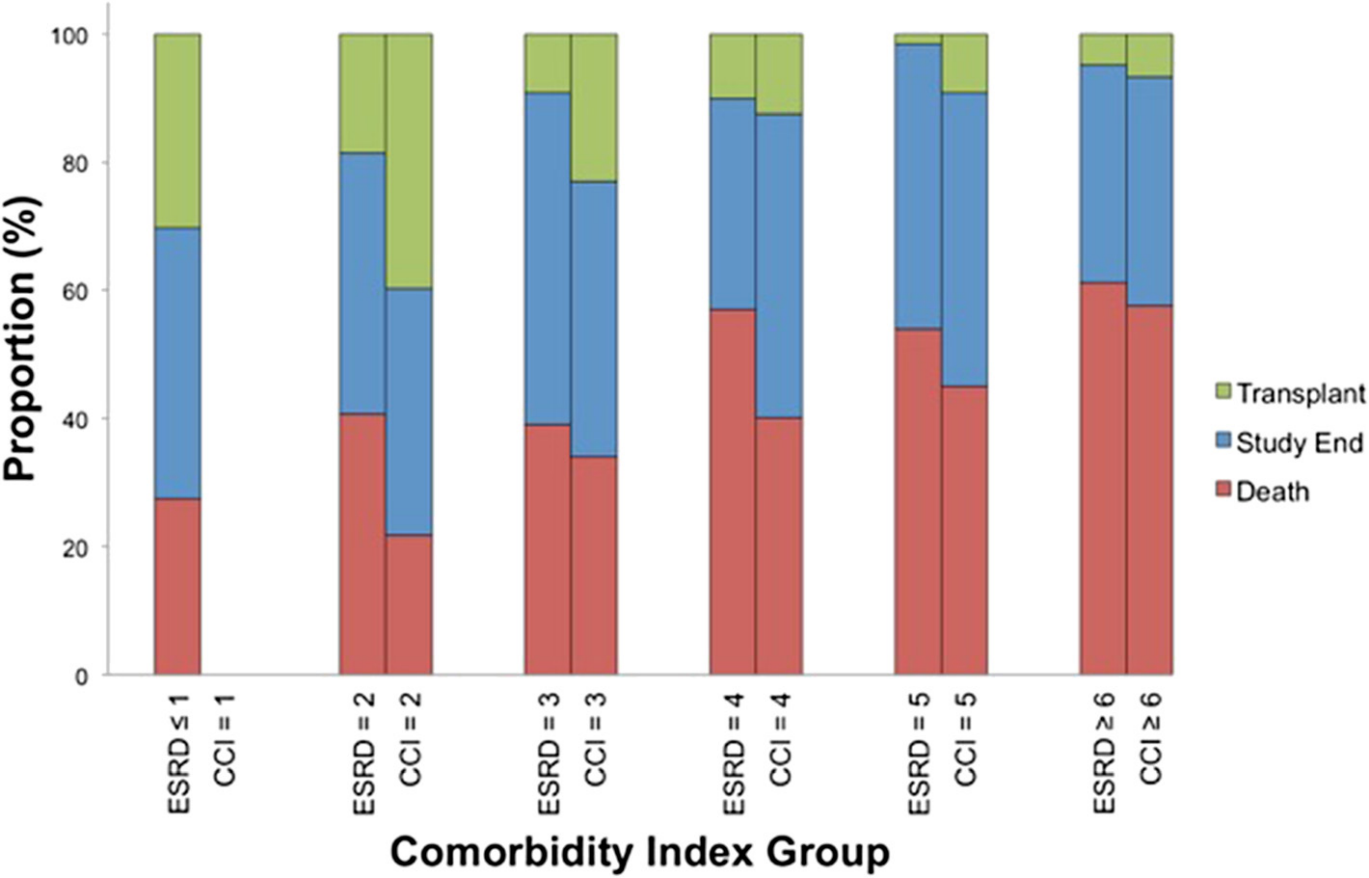
Distribution of outcomes stratified by Comorbidity Index Score groups

Over 1796.6 patient years at risk, there were 311 deaths. The distribution of deaths stratified by CCI and ESRD-CI scores are graphically displayed in Fig. 3. There was a rise in the number of deaths and fall in the number of patients that received a kidney transplant with each ESRD-CI score cut-off. In an unadjusted Cox survival analysis, relative to patients with an ESRD-CI score of ≤1, those with scores of ≥6 had a mortality HR of 2.64 (95 % CI 1.91 to 3.65, p < 0.001, Table 3). A similar mortality HR was observed for patients with CCI scores of ≥6 compared to 2 (HR 2.91, 95 % CI 2.04 to 4.15, p < 0.001). After multivariable adjustment, there was attenuation in the HR. Similar HR’s were noted for patients with scores of 4–6 for the ESRD-CI. For the CCI, there was separation in the HR’s for scores of 4–6 however, confidence intervals overlapped (Table 3).

**Table 3:** Multivariable Cox survival analysis for the ESRD-CI and CCI

Model	ESRD-CI Group	Events (N)	HR [95 % CI]	Model	CCI Group	Events (N)	HR [95 % CI]
Unadjusted	≤1 2	311	(ref) 1.74 [1.22 to 2.50]	Unadjusted	1 2	311	--- (ref)
	3		1.39 [0.94 to 2.07]		3		1.84 [1.17 to 2.90]
	4		2.32 [1.62 to 3.32]		4		1.83 [1.23 to 2.72]
	5		2.37 [1.60 to 3.52]		5		2.31 [1.54 to 3.47]
	≥6		2.64 [1.91 to 3.65]		≥6		2.91 [2.04 to 4.15]
Model 1^a^	≤1 2	311	(ref) 1.60 [1.12 to 2.30]	Model 1^a^	1 2	311	--- (ref)
	3		1.19 [0.80 to 1.76]		3		1.64 [1.04 to 2.59]
	4		2.00 [1.40 to 2.86]		4		1.66 [1.12 to 2.47]
	5		1.97 [1.32 to 2.93]		5		2.12 [1.41 to 3.18]
	≥6		2.12 [1.52 to 2.93]		≥6		2.44 [1.71 to 3.49]
Model 2^b^	≤1 2	311	(ref) 1.62 [1.13 to 2.33]	Model 2^b^	1 2	311	--- (ref)
	3		1.21 [0.81 to 1.80]		3		1.66 [1.05 to 2.63]
	4		2.02 [1.41 to 2.90]		4		1.69 [1.14 to 2.52]
	5		1.99 [1.33 to 2.96]		5		2.13 [1.42 to 3.21]
	≥6		2.11 [1.52 to 2.93]		≥6		2.47 [1.73 to 3.53]
Model 3^c^	≤1 2	311	(ref) 1.60 [1.11 to 2.30]	Model 3^c^	1 2	311	--- (ref)
	3		1.21 [0.80 to 1.81]		3		1.64 [1.03 to 2.59]
	4		2.02 [1.40 to 2.93]		4		1.86 [1.23 to 2.81]
	5		1.91 [1.27 to 2.90]		5		2.29 [1.48 to 3.54]
	≥6		2.09 [1.49 to 2.93]		≥6		2.75 [1.86 to 4.08]
Model 4^d^	≤1 2	311	(ref) 1.57 [1.09 to 2.27]	Model 4^d^	1 2	311	--- (ref)
	3		1.20 [0.80 to 1.80]		3		1.63 [1.03 to 2.59]
	4		1.97 [1.36 to 2.87]		4		1.83 [1.21 to 2.77]
	5		1.86 [1.23 to 2.81]		5		2.24 [1.44 to 3.47]
	≥6		2.06 [1.46 to 2.89]		6		2.70 [1.81 to 4.02]
Model 5^e^	≤1 2	309	(ref) 1.63 [1.12 to 2.36]	Model 5^e^	1 2	309	--- (ref)
	3		1.28 [0.84 to 1.91]		3		1.76 [1.10 to 2.82]
	4		1.95 [1.34 to 2.85]		4		1.86 [1.22 to 2.83]
	5		1.89 [1.25 to 2.86]		5		2.38 [1.53 to 3.72]
	≥6		1.99 [1.41 to 2.81]		≥6		2.71 [1.81 to 4.06]

^a^: Adjusted for age

^b^: Adjusted for factors in a., race and gender

^c^: Adjusted for factors in b. and cause of ESRD

^d^: Adjusted for factors in c. and dialysis type

^e^: Adjusted for factors in d. and laboratory data (MDRD GFR, albumin, phosphate, hemoglobin)

## Discussion

In this cohort study we evaluated the ability of the CCI and ESRD-CI to predict mortality in a population of incident dialysis patients. The association between comorbidity and mortality in our dataset was not as strong as in the derivation cohorts. In addition although higher comorbidity burden using the CCI and ESRD-CI was associated with mortality after multivariable adjustment, there was not a large separation in mortality risk when evaluating incremental changes in comorbidity scores. We can speculate that the limited utility of the CCI and ESRD-CI is due to several potential underlying reasons; limited generalizability of both indices, variability in comorbidity classification and comorbidity prevalence within either cohort, limitations with respect to derivation (including statistical over-fitting) and limited utility of comorbidity indices in general.

Limited generalizability may explain the observation of only partial validity. The CCI and ESRD-CI used a relatively small population from a single geographical area and validation in this study occurred in a separate single geographical area. Differences in the determinants of health in two communities may contribute to the different outcomes of two dialysis patients with similar comorbidities. Social determinants of health, in particular, have been shown to impact mortality rates in patients with ESRD [37–39]. More recently, frailty has been shown to be an important prognostic factor for incident dialysis patients [40]. Therefore, prediction models that incorporate clinical, demographic and social factors as well as assessments of frailty may be more applicable to ESRD cohorts [41]. An attempt at further validation of this index in a national or international sample of patients from a number of different centers in different geographic regions might help to clarify its generalizability and provide a clearer picture of its clinical utility.

There were notable characteristic differences comparing patients in our dataset and the dataset of the original studies, which may explain the reduced discrimination. Our cohort was in a more recent era (2006–2013), and the relative impact of some comorbid conditions may have changed [42–45]. The mortality risk in our cohort was higher than either derivation cohort [15] however; certain comorbidities such as chronic lung disease (16.7 % vs. 27.4 %) and neoplasia (6.5 % vs. 12.2 %) were less common in our cohort [15]. Furthermore, there was a reduced prevalence of overall comorbidity compared to the derivation cohorts. This reduced burden of disease might have affected the predictive value of the individual comorbidities comprising both indices. Alternatively, under-reporting of individual health conditions in each cohort may have explained the observed differences. For example, if comorbidity in our validation cohort was under-reported, despite a “falsely low” burden of comorbidity patients would have continued to have a high mortality rate. This in turn would have reduced the discrimination in our dataset.

The derivation of the CCI or ESRD-CI may have also impacted its validation. The linear predictor from a Cox model is ideal for developing a prognostic index. The linear predictor is described as the weighted sum of the variables in the model, where the regression coefficients are the weights [25]. In the development of the CCI, (which was replicated in the derivation of the ESRD-CI to maintain consistency) scoring weights were assigned to each HR derived from the Cox model [4, 15]. This may lead to over-weighting of conditions with high hazard ratios but limited precision and marked variability around the estimate.

While there are limitations with the comorbidity indices, it is important to acknowledge that both indices did have some validity. It is not unexpected that a higher level of comorbidity would be associated with a higher risk of death among dialysis patients, however most evaluations of comorbidity scores look at short term mortality or use validation techniques that do not incorporate survival time [15, 19, 23, 24]. Discrimination was lower in our validation dataset, however, the relative hazard for death was proportional across the index and the mortality association using either index persisted despite a relatively long duration of follow-up. Furthermore, it is not uncommon for validation studies to identify some reduction in predictive value [46] In addition there are other features of these indices that make them valuable. Both are intuitive; comorbid conditions that would be expected to confer a higher hazard for mortality are weighted more heavily. The major exception to this would be HIV (a component of the original CCI) a condition with a lower contemporary mortality rate [42]. Another advantage is that both indices draw on clinical data that is often present in patient charts, making them practical tools that do not necessarily require other diagnostic testing or laboratory investigation. Finally, the association with mortality persisted in our study even after multivariable adjustment for known predictors of mortality among dialysis patients.

It is important to note that there are limitations of comorbidity indices in general that warrant consideration. Most indices do not fully take into account the stage of progression, severity or proximity of the comorbid condition in relation to dialysis initiation [1, 14–16]. Furthermore, accumulation of comorbidity that often accompanies the early period after dialysis initiation [47] is not typically included in comorbidity indices. Global scoring systems that incorporate all prior comorbidities are easier to calculate and extract from patient records and facilitate ease of clinical application at point-of-care. More novel indices such as the Adult Comorbidity Evaluation-27, which takes into account the proximity and severity of the comorbidity, have been shown to out perform the CCI at predicting mortality [48, 49] and may be better suited for dialysis cohorts. Additionally, simple prognostic models that include the “surprise question” have also been shown to accurately predict survival for patients receiving hemodialysis [50].

This study utilized a substantial Canadian cohort and a long follow up time while leveraging electronic medical records to ensure a high quality and robust dataset. Additionally validation of the index was performed utilizing stringent methodology [25]. Examining the adjusted association between the CCI and ESRD-CI and mortality was enhanced by the relatively large number of outcomes.

There are, however, limitations to this study. The classification of comorbidities for each patient was determined by clinical impression (based on documentation in paper chart and electronic records). This introduces the possibility for misclassification bias. The derivation cohort(s) would have also been affected by misclassification bias, potentially compounding the observed differences in disease prevalence and prognostic utility. Our ability to completely validate either index was limited by the level of information provided in the original studies. In particular, an assessment of calibration (the ability of the index to assign the correct event probability at any relevant follow-up time and every level of predicted risk [25]) could not be performed.

## Conclusion

The CCI and ESRD-CI had a limited ability to discriminate risk of death for incident dialysis patients in a contemporary Canadian cohort. Although a higher comorbidity burden was associated with mortality, incremental increases in index scores did not considerably change the risk of death.
